# *In Situ* Normoxia Enhances Survival and Proliferation Rate of Human Adipose Tissue-Derived Stromal Cells without Increasing the Risk of Tumourigenesis

**DOI:** 10.1371/journal.pone.0115034

**Published:** 2015-01-23

**Authors:** Jane Ru Choi, Belinda Pingguan-Murphy, Wan Abu Bakar Wan Abas, Kar Wey Yong, Chi Tat Poon, Mat Adenan Noor Azmi, Siti Zawiah Omar, Kien Hui Chua, Feng Xu, Wan Kamarul Zaman Wan Safwani

**Affiliations:** 1 Department of Biomedical Engineering, Faculty of Engineering, University of Malaya, Lembah Pantai, 50603 Kuala Lumpur, Malaysia; 2 Department of Obstetrics and Gynaecology, Faculty of Medicine, University of Malaya, Lembah Pantai, 50603 Kuala Lumpur, Malaysia; 3 Department of Physiology, Faculty of Medicine, Universiti Kebangsaan Malaysia, Jalan Raja Muda Abdul Aziz, 50300 Kuala Lumpur, Malaysia; 4 The Key Library of Biomedical Information Engineering of Ministry of Education, School of Life Science and Technology, Xi’an Jiaotong University, Xi’an 710049, P.R. China; 5 Bioinspired Engineering and Biomechanics Center (BEBC), Xi’an Jiaotong University, Xi’an 710049, P.R. China; French Blood Institute, FRANCE

## Abstract

Adipose tissue-derived stromal cells (ASCs) natively reside in a relatively low-oxygen tension (i.e., hypoxic) microenvironment in human body. Low oxygen tension (i.e., *in situ* normoxia), has been known to enhance the growth and survival rate of ASCs, which, however, may lead to the risk of tumourigenesis. Here, we investigated the tumourigenic potential of ASCs under their physiological condition to ensure their safe use in regenerative therapy. Human ASCs isolated from subcutaneous fat were cultured in atmospheric O_2_ concentration (21% O_2_) or *in situ* normoxia (2% O_2_). We found that ASCs retained their surface markers, tri-lineage differentiation potential, and self-renewal properties under *in situ* normoxia without altering their morphology. *In situ* normoxia displayed a higher proliferation and viability of ASCs with less DNA damage as compared to atmospheric O_2_ concentration. Moreover, low oxygen tension significantly up-regulated VEGF and bFGF mRNA expression and protein secretion while reducing the expression level of tumour suppressor genes p16, p21, p53, and pRb. However, there were no significant differences in ASCs telomere length and their relative telomerase activity when cultured at different oxygen concentrations. Collectively, even with high proliferation and survival rate, ASCs have a low tendency of developing tumour under *in situ* normoxia. These results suggest 2% O_2_ as an ideal culture condition for expanding ASCs efficiently while maintaining their characteristics.

## Introduction

Stem cells have attracted significant interest as a cell source for regenerative medicine and cell therapy due to their differentiation and self-renewal capacity [1–3]. Among various types of stem cells, adipose tissue-derived stromal cells (ASCs) have recently become particularly attractive due to the relative abundance and accessibility of adipose tissue [4–6]. Interestingly, despite the fact that adipose tissue is highly vascularised [7], they have been found residing in a native hypoxic microenvironment [8, 9], *e.g*., 3% oxygen concentration in mouse adipose tissue [10] and <4% O_2_ in human adipose tissue [11, 12]. However, oxygen tension is often overlooked when performing *in vitro* study of stem cells [13, 14], where 21% oxygen concentration is normally used for culture of ASCs. Therefore, it is essential to understand the response of ASCs to in situ normoxia for developing cell-based therapy strategies.

As large numbers of ASCs are required for clinical application, many studies have emphasized the effect of in situ normoxia on cell expansion. Most studies have demonstrated that human ASCs displayed a higher proliferation and survival rate at low oxygen concentration as compared to normal oxygen concentration [15–17]. It has been reported that the release of hypoxia inducible factor-1 alpha (HIF-1α) under low oxygen tension increased the expression of growth factors, such as vascular endothelial growth factor (VEGF), basic fibroblast growth factor (bFGF), epidermal growth factor (EGF), transforming growth factors (TGF), insulin-like growth factor (IGF), hepatocyte growth factor (HGF) and platelet-derived growth factor (PDGF) [12, 18–20]. These growth factors might in turn promote the growth and survival of the cells [21]. However, a high rate of proliferation and an anti-apoptotic characteristic resembles the behaviour of cancerous cells, suggesting the potential of malignant transformation and cancer development [22, 23]. Conversely, it has been suggested that culture of ASCs at low oxygen concentration may enhance their genetic stability [15]. To our knowledge, the tumourigenic potential of ASCs under in situ normoxia has not yet been well investigated.

Notably, 2% O_2_ was most frequently reported to enhance the expansion and survival rate of ASCs [16, 17, 24–26]. Thus, a biosafety assessment should be performed prior to clinical use. There has been controversy in research findings regarding the tumorigenic potential of MSCs. Some groups have reported that human MSCs may develop tumourigenesis after long-term culture (eg. beyond 5 weeks [27–30]). However, recent studies have shown that MSCs can be expanded over multiple cell doublings (3–6 months) without chromosomal alterations, demonstrating a low risk of tumourigenesis [31–33]. In this study, we aimed to determine the tumourigenic potential of ASCs cultured at 2% oxygen tension at early passage. We first characterized the ASCs by examining their morphology, differentiation capabilities, surface markers expression, and self-renewal properties under both atmospheric O2 concentration (21% O_2_) and in situ normoxia (2% O_2_). We then investigated the influence of in situ normoxia on viability and proliferation rate of ASCs, followed by determining the HIF-1α expression level and the production of growth factors under this physiological condition. Thereafter, we evaluated the tumourigenic potential of ASCs in terms of tumour suppressor gene expression level, alteration in telomere length and telomerase activity as well as the degree of DNA damage.

## Materials and Methods

### Ethics Statement

This study was approved by the Medical Ethics Committee of University Malaya Medical Centre (UMMC). Subcutaneous adipose tissue was collected from female donors aged 25 to 45 undergoing caesarean section with the informed written consent under a protocol approved by the Medical Ethics Committee of UMMC (reference no. 996.46). Consent was obtained from each donor before collecting the samples.

### Isolation and culture of human adipose tissue-derived stromal cells

The adipose tissue was washed with phosphate buffered saline (PBS) (Sigma-Aldrich, St Louis, MO, USA), minced and digested with 0.3% collagenase I (Worthington, Freehold, NJ, USA). The digested tissue was washed with PBS and centrifuged. The pellet was cultured with Dulbecco’s Modified Eagle’s Medium (DMEM)/Ham F-12 growth medium containing 10% FBS, 1% vitamin C, 1% glutamax, and 1% antibiotic-antimycotic solution (Gibco, Grand Island, NY, USA) in culture flasks. As a normal control group, the cells were cultured with 21% O_2_ and 5% CO_2_ at 37°C. The culture media was then replaced every 2–3 days. ASCs at passage 3 were used for all the tests unless otherwise stated.

### *In situ* normoxia

ASCs were cultured at 37°C in an O_2_ incubator (Galaxy 170 R, New Brunswick Scientific, USA). The incubator was supplied with 2% O_2_ and 5% CO_2_ whereby low O_2_ concentration was maintained with nitrogen gas (N_2_) supply. The oxygen level was confirmed with Jenway 970 portable dissolved oxygen meter (Bibby Scientific Limited, Staffordshire, UK). Similar to the controls (cells cultured under atmospheric O2 concentration), the culture media under in situ normoxia was replaced every 2–3 days. ASCs at passage 3 were tested immediately after they had been taken out from the incubator.

### ASC characterization

To make sure that the cells used in this experiment are mesenchymal stem cells (MSCs), we followed the three standard criteria suggested by Dominici *et al*. [34], stating that, firstly MSCs must be plastic-adherent. Secondly, they must express their surface markers CD73, CD90 and CD105 while showing negative for haematopoetic markers CD14, CD34 and CD45, B cell marker CD 19, and histocompatibility class II cell surface receptor HLA-DR, DP, DQ which are found only on antigen-presenting cells and B cells. Additional histocompatibility class I cell surface receptor, HLA-ABC was also tested in this study which is typically expressed by MSCs. Thirdly, MSCs must possess the potential to differentiate into adipocytes, osteoblasts and chondroblasts [34].

In this study, the morphology of the cells was examined, followed by determining the expression of their surface markers and their capability of differentiation. Flow cytometry was performed to identify the ASCs surface markers under in situ normoxia and atmospheric O2 concentration. ASCs from both groups were harvested, washed and immuno-stained with FITC-conjugated antibodies against CD105, CD90, CD45, CD34, HLA-ABC and HLA-DR, DP, DQ and PE-conjugated antibodies against CD73, CD14 and CD19 (Becton Dickinson, San Jose, CA, USA). Negative control staining was done using FITC-conjugated mouse IgG1 & IgG2 isotypes, and PE-conjugated mouse IgG1 & IgG2 isotypes (Becton Dickinson). Flow cytometry was conducted using BD FACSCanto II (Becton Dickinson). A minimum of 10000 events for each sample were acquired and analysed with FlowJo analysis software (Treestar, Ashland, OR, USA).

For adipogenic and osteogenic differentiation, ASCs at passage 3 were initially grown in petri dishes with growth medium which was then replaced with their corresponding induction medium when they reached confluence. Adipogenic induction medium contained high glucose DMEM supplemented with 10% FBS (Gibco), 0.5 μM isobutyl-1-methyl xanthine, 1 μM dexamethasone and 200 μM indomethacin (Sigma-Aldrich). Osteogenic induction medium contained high glucose DMEM with 10% FBS (Gibco), 0.05 mM ascorbic acid-2-phosphate, 10 mM b-glycerophosphate and 100 nM dexamethasone (Sigma-Aldrich). Differentiation medium was changed every 3 days for up to 21 days, with the cells cultured in both atmospheric O2 concentration and in situ normoxia. Adipogenic differentiation was confirmed by detecting the presence of lipid droplets stained with Oil red O (Sigma-Aldrich), whereas osteogenenic differentiation was assessed by staining of calcium deposits using Alizarin red (Sigma-Aldrich). For chondrogenic differentiation, ASCs at passage 3 were pelleted and cultured in chondrogenic induction medium composed of DMEM/F12 supplemented with 1% FBS, 1% glutamax, 1% vitamin C, 1% antibiotic-antimycotic (Gibco), ITS premix (Becton Dickinson), 40μg/ml L-proline, 100nM dexamethasone, 50 μg/ml ascorbate- 2-phophate (Sigma-Aldrich), 50 ng/ml IGF-1 and 10 ng/ml TGF-β1 (Peprotech, Rocky Hill, NJ, USA). Culture medium was changed every 3 days for 21 days. For histological examination, briefly, pellets were fixed overnight in 10% formalin (Sigma-Aldrich) and processed according to the standard procedures for sample processing, embedding and sectioning. Proteoglycans accumulation was assessed using alcian blue staining (Sigma-Aldrich). The stained sections were mounted with mounting medium (DPX) (Sigma-Aldrich) and visualized with a light microscope (Eclipse TS100, Nikon, NY, USA).

### Colony-forming unit-fibroblast (CFU-F) assays

ASCs at P3 were plated at a density of 400 cells/ cm^2^ in 100-mm culture dishes under in situ normoxia or atmospheric O2 concentration. The medium was changed every 3 days for a period of 14 days. The culture dishes were rinsed with PBS, fixed with 10% formalin solution and stained for 20 min with Giemsa stain (Sigma-Aldrich). After rinsing with PBS, colonies consist of more than 50 stained cells were counted. Total colony numbers was determined.

### Cell proliferation assays

Cell proliferation was assessed by Resazurin reduction assay and population doubling time. Briefly, about 140 mg Resazurin sodium salts (Sigma-Aldrich) was dissolved in 1 L PBS (Sigma-Aldrich, USA) to prepare Resazurin stock solution. Resazurin 10% working solution was then prepared from the stock solution. ASCs were seeded onto a 24-well plate at 5 × 10^4^ cells per well with growth media on day 0. Resazurin assay was performed on day 1, 3, 7, 10, 14, 17 and 21. The absorbance signals were quantified at excitation wavelength of 570 nm and emission wavelength of 595nm, using a FLUOstar Optima microplate reader (BMG Labtech, Offenburg, Germany). Viable cells were quantified as percentage of Resazurin reduction. At the same time, cell suspensions were serially diluted and seeded into a 24-well plate. After 24 hours of incubation, the percentage of Resazurin reduction was determined following the steps explicitly mentioned above. The relationship between Resazurin reduction (%) and cell numbers was determined by plotting a standard curve. A growth curve was then generated by plotting a graph of cell number versus time to compare the growth rate between both groups.

The growth kinetic was evaluated to further determine the proliferation ability of ASCs at different conditions. This was determined by population doubling time (PDT, time required for a culture to double in number). Cells were cultured under two different conditions until passage 3 and the number of cells was determined at the beginning and the end of each passage. Number of cell doublings were calculated based on the formula n = (log_10_ N_h_- log_10_ N_i_) /log_10_ 2, where N_h_ is the cell number at the end of passage and N_i_ is the cell number at initial seeding. Population doubling time was calculated as a ratio of number of days in culture divided by the number of cell doublings at each passage. Mean PDT of 3 passages was then determined.

### Cell cycle analysis

Samples for cell cycle analysis were prepared using CycleTEST Plus DNA Reagent Kit (Becton Dickinson) in accordance with the manufacturer’s instruction. Flow cytometry was performed using *BD Accuri* C6 (Becton Dickinson). The percentage of cells in G0/G1, S and G2/M phases of cell cycle was determined using ModFit LT 4.0 software (Verity Software House, Topsham, ME, USA).

### Apoptosis assay

The apoptosis assay was performed using Annexin V: FITC Apoptosis Detection Kit I (Becton Dickinson) following the manufacturer’s instructions. Flow cytometry analysis was carried out using *BD Accuri* C6 (Becton Dickinson).

### Alkaline comet assay

The comet assay was conducted using alkaline conditions according to the manufacturer’s instruction of Comet Assay Kit (Trevigen, Gaitherburg, MD, USA). After staining with ethidium bromide (Invitrogen, Carlsbad, CA, USA), images (magnification 100x) were observed and captured using inverted fluorescence microscope (ECLIPSE TI-S, Nikon, NY, USA) connected to a digital camera. About 100 nuclei per sample were analyzed using Comet Assay IV 4.3 software (Perspective Instrument, Haverhill, UK). The three parameters: percentage of DNA in the tail, tail length and tail moment were used to evaluate the DNA damage.

### RNA extraction, cDNA synthesis and quantitative real-time polymerase chain reaction (qPCR)

RNA extraction was performed using TRI reagent (Ambion, Austin, TX, USA), followed by phase separation with chloroform (Fisher Scientific, Loughborough, UK) and RNA precipitation with isopropanol (Sigma-Aldrich). The high capacity RNA-to-cDNA kit (Applied Biosystems, Foster City, CA, USA) was used to synthesize cDNAs. Then qPCR was conducted with TaqMan gene expression assays (Applied Biosystems) using StepOnePlus Real-Time PCR system (Applied Biosystem). The genes tested include HIF-1a (Hs00153153_m1), TP53 (Hs00153349_m1), CDKN1A (Hs00355782_m1), CDKN2A (Hs00923894_ml), TERT (Hs00972656_ml), RB1 (Hs01078066_ml), VEGFA (Hs00173626_ml), PDGFA (Hs00234994_ml), FGF2 (Hs00266645_ml), HGF (Hs00300159_ml), IGF1 (Hs01547656_ml), EGF (Hs01099999_ml), TGFB1 (Hs00171257_ml) and GAPDH (Hs99999905_ml). GAPDH was used as housekeeping gene for normalization. The thermal cycling conditions include incubation for 20 s at 95°C to activate AmpliTaq Fast DNA polymerase, followed by 40 cycles of denaturation for 1 s at 95°C, annealing and elongation for 20 s at 60°C. The gene expression of control group (atmospheric O2 concentration) was normalized to 1. The results were expressed as fold changes in gene expression relative to the control.

### ELISA

As VEGF and bFGF gene expression level were significantly higher under in situ normoxia than atmospheric O2 concentration as indicated by qRT-PCR, their production were quantified using human Quantikine ELISA kit (R&D Systems, Minneapolis, MN, USA) following the manufacturer’s instructions.

### Telomerase assay

Telomerase activity of ASCs was determined using the TeloTAGGG PCR ELISA PLUS kit (Roche, Mannheim, Germany), based on telomere repeat amplification protocol (TRAP) according to the manufacturer’s recommendations. The relative telomerase activities (RTA) were determined using the formula provided by the manufacturer.

### Telomere length analysis

Genomic DNA was extracted from ASCs cultured in two different conditions using the PureLink Genomic DNA Mini Kit (Invitrogen). Telomere length analysis was carried out using TeloTAGGG Telomere Length Assay kit (Roche) in accordance with the manufacturer’s instructions. The chemiluminescence was detected by gel documentation imaging system (Fusion FX5, Vilber Lourmat, France). By comparing the signal relative to a molecular weight standard, the mean telomere restriction fragment (TRF) length was quantified.

### Statistical analysis

Student’s *t* test was used to compare the normally distributed data between in situ normoxia and atmospheric O2 concentration. Data were expressed as mean ± standard error of the mean (SEM) of six different experiments with six different donors (n = 6). p < 0.05 was reported as statistically significant.

## Results

### ASCs retain their characteristics under *in situ* normoxia

**Figure 1:**
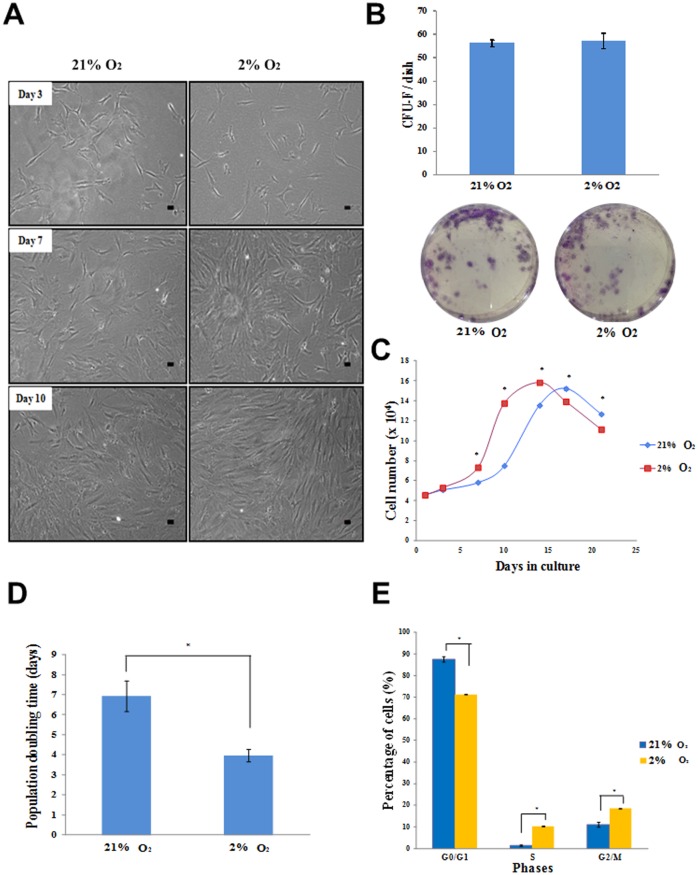
In situ normoxia enhanced the expansion rate of ASCs without changing their morphology and self-renewal properties. Cells maintained fibroblast-like appearance on day 3, 7 and 10 of culture. The density of the cells was remarkably increased under in situ normoxia, magnification 100x (A). In situ normoxia maintained the number of CFU-F. Macroscopic examination showed the single colonies of ASCs, representing the population which exhibits the self-renewal properties of MSC (B). Growth curve of ASCs (C) showed an increased rate of cell proliferation under in situ normoxia. Population doubling time (D) of ASCs was significantly lower under in situ normoxia than in atmospheric O2 concentration. Cell cycle analysis showed the percentage of ASCs in S-phase was higher under in situ normoxia as compared to atmospheric O2 concentration (E). Data shown represent as mean ± SEM. * indicates p < 0.05 relative to atmospheric O2 concentration.

To determine if ASCs are able to maintain their characteristics under in situ normoxia, their morphological feature was first observed. Under both in situ normoxia and atmospheric O2 concentration, ASCs displayed adherent fibroblast-like features. There were no morphological changes from day 1 to day 10 of the culture (Fig. 1A). In addition to morphological examination, ASCs were further characterized through the expression of their surface markers and their differentiation capabilities. The result of flow cytometry analysis showed no significant differences in ASCs surface marker expression at different oxygen tensions. They were positive for the stem cell surface markers CD73, CD90, CD105 and HLA-ABC, and negative for HLA-DR DP DQ, CD14, CD19, CD34 and CD45, indicating that in situ normoxia did not alter the phenotype of ASCs (S1 Fig.). Moreover, like atmospheric O2 concentration, in situ normoxia also maintained the adipogenic, osteogenic and chondrogenic differentiation abilities of ASCs, as indicated by the positive staining (S2 Fig.). Additionally, to show the presence of cells exhibiting the functional properties of MSC, we performed colony-forming unit-fibroblast (CFU-F) assay. Our result showed the formation of single colonies of ASC, representing the cell population with self-renewal properties. There was no significant difference in the number of colonies in both groups, indicating the maintenance of the stemness properties of ASC under in situ normoxia (Fig. 1B).

### *In situ* normoxia enhances the expansion rate of ASCs

To determine if the cells grow more rapidly in their physiological condition, we measured the proliferation rate of ASCs at different oxygen tensions. Even though there was no alteration in their morphology, we observed a remarkable increase in cell density under in situ normoxia from day 7 to day 10 as compared to atmospheric O2 concentration (Fig. 1A). This indicates an increased proliferation rate of ASCs under in situ normoxia. To further quantify the growth rate of cells, Resazurin reduction assay and population doublings time were assessed. We found an increased number of ASCs under in situ normoxia as compared to atmospheric O2 concentration from day 7 of the culture. Specifically, at day 10, ASCs cultured under in situ normoxia had a 1.8-fold greater cell number (13.75 × 10^4^ ± 0.09 × 10^4^) than atmospheric O2 concentration (7.5 × 10^4^ ± 0.039 × 10^4^) (Fig. 1C). Additionally, at 2% O_2_, ASCs displayed a lower population doubling time as compared to atmospheric O2 concentration with the mean population doubling time of 3.97 ± 0.31 days and 6.93 ± 0.75 days, respectively, p < 0.05 (Fig. 1D).

To confirm the result of cell proliferation, cell cycle analysis was carried out by flow cytometry. The result showed that the average percentage of cells in G0/G1 phase was significantly higher in atmospheric O2 concentration (87.59 ± 1.27%) than under in situ normoxia (71.23 ± 0.095%), whereas the fraction of cells in S phase was higher under in situ normoxia (10.32 ± 0.14%) than atmospheric O2 concentration (1.38 ± 0.25%), p < 0.05 (Fig. 1E).

### *In situ* normoxia enhances ASCs viability and reduces DNA damage

**Figure 2:**
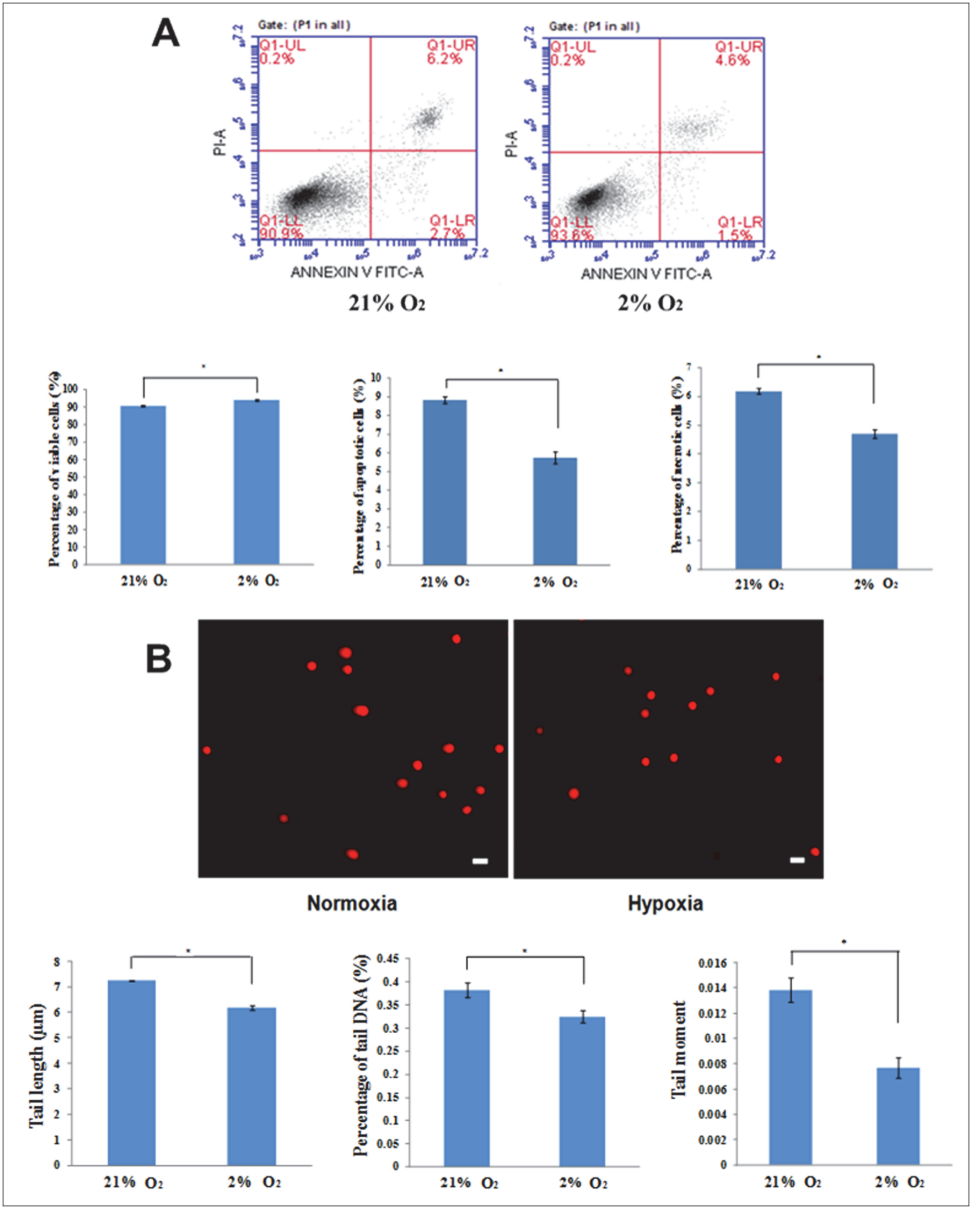
In situ normoxia enhanced the survival of ASCs by reducing the rate of apoptosis and DNA damage. In situ normoxia showed a higher percentage of viable cells with lower numbers of apoptotic and necrotic cells than atmospheric O2 concentration (A). A representative figure of DNA showed more cells without comet tails were observed under in situ normoxia, magnification 100x. Arrows show the comet tails. ASCs cultured under in situ normoxia displayed a significantly lower tail length, percentage of tail DNA and tail moment in comparison with ASCs under atmospheric O2 concentration (B). Each data represents mean ± SEM. * indicates p < 0.05 relative to atmospheric O2 concentration.

To assess the viability of ASCs, we carried out Annexin V/propidium iodide apoptosis assay. With the combination of both Annexin V+ and PI+ labelling, the percentage of viable cell in atmospheric O2 concentration was significantly lower (90.48 ± 0.35%) than that of in situ normoxia (93.9 ± 0.31%), p < 0.05 (Fig. 2A).

To confirm the level of DNA damage under in situ normoxia, comet assays were performed. The cells cultured under in situ normoxia displayed a significant shorter tail length (6.18 ± 0.1μm vs 7.23 ± 0.02 μm in the ASC under atmospheric O2 concentration, p < 0.05), lowered % DNA in the tail (0.32 ± 0.01% vs 0.38 ± 0.02% in the ASC under atmospheric O2 concentration, p < 0.05) and lowered tail moment (0.008 ± 0.0008 vs 0.014 ± 0.001 in the ASC under atmospheric O2 concentration, p < 0.05) as compared with that of atmospheric O2 concentration (Fig. 2B).

### *In situ* normoxia increases HIF-1α expression, VEGF-A and bFGF secretion

**Figure 3:**
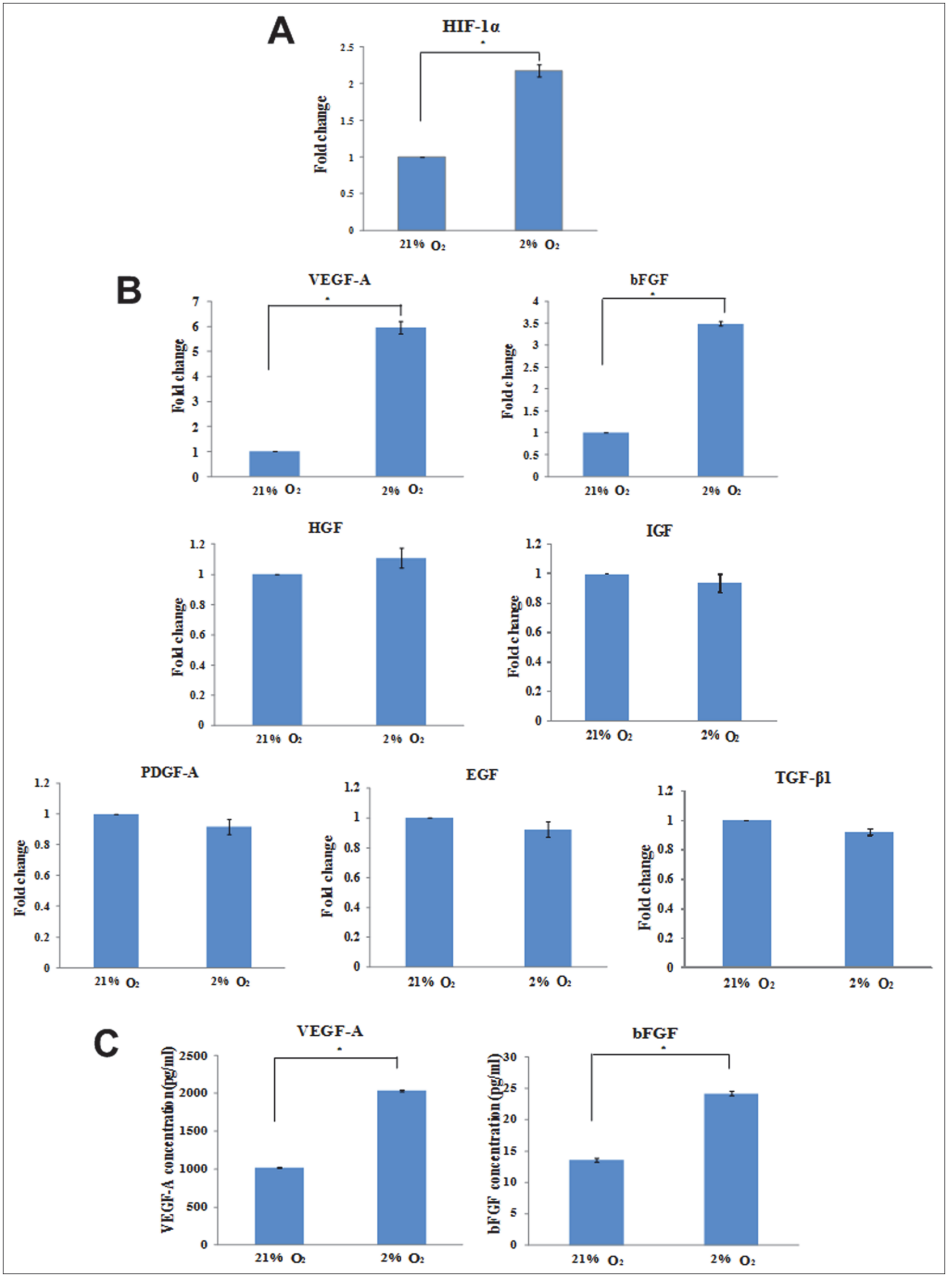
Effects of in situ normoxia on HIF-1α expression and both expression and secretion of growth factors. The expression of HIF-1α was significantly higher under in situ normoxia than in atmospheric O2 concentration (A). In situ normoxia displayed a significant higher expression level of VEGF-A and bFGF as compared to atmospheric O2 concentration (B). The secretion of VEGF-A and bFGF were also increased under in situ normoxia (C). Data are presented as mean ± SEM. * indicates p < 0.05 relative to atmospheric O2 concentration.

To investigate if in situ normoxia induces the expression of HIF-1α and several growth factors which might be involved in proliferation of ASCs, we performed RT-PCR. Gene expression studies revealed that the HIF-1α expression was significantly higher under in situ normoxia than in atmospheric O2 concentration (Fig. 3A). The expression of HIF-1 downstream target genes, VEGF-A and bFGF were significantly increased 6 fold and 3.5 fold, respectively, under in situ normoxia as compared to atmospheric O2 concentration. However, the expression levels of other growth factors, PDGF, HGF, IGF, EGF and TGF remained unchanged when exposed to in situ normoxia (Fig. 3B).

To confirm the translation of VEGF-A and bFGF mRNAs to proteins, ELISA was performed. In concordance with gene expression levels, ELISA showed that ASCs under in situ normoxia significantly increased the secretion of VEGF-A (2-fold) as compared to ASCs under atmospheric O2 concentration (2036.35 ± 12.24 vs 1016.97 ± 6.47 pg/ml, p<0.05). Similar result was shown in bFGF, where their secretion was significantly increased in ASCs under in situ normoxia (24.18 ± 0.33 pg/ml) compared to ASCs under atmospheric O2 concentration (13.59 ± 0.32 pg/ml), representing a 1.8–fold increase (Fig. 3C).

### *In situ* normoxia demonstrates low risk of tumourigenesis

**Figure 4:**
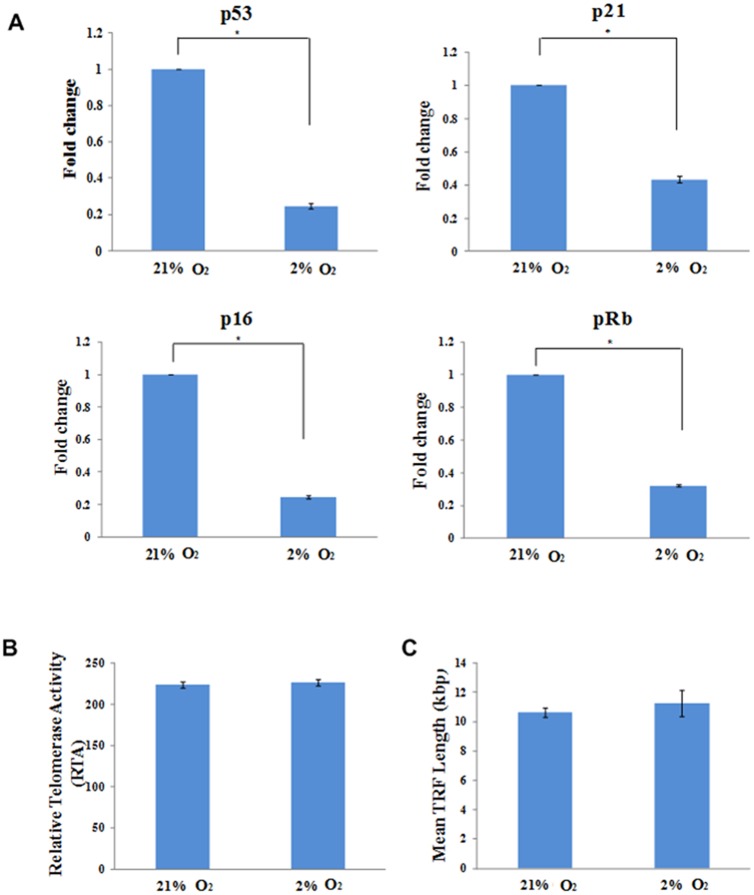
In situ normoxia lowered the expression of tumour suppressor genes and maintained both telomerase activity and TRF length of ASCs. In situ normoxia displayed lower expression levels of p53, p21, p16 and pRb (A) as compared to atmospheric O2 concentration. There were no significant difference between both groups on their telomerase activity (B) and mean TRF length (C). Data are presented as mean ± SEM. * indicates p < 0.05 relative to atmospheric O2 concentration.

To investigate the tumourigenic potential of ASCs, we determined the expression levels of tumor suppressor genes pRb, p16, p21 and p53, which are involved in tumour suppressor pathways that regulate cellular responses to stress stimuli. We found that the expression of tumour suppressor genes pRb, p16, p21 and p53 were significantly lower under in situ normoxia than atmospheric O2 concentration, suggesting that in situ normoxia down-regulates tumour suppressor activity (Fig. 4A).

To further assess the risk of tumourigenesis, the telomerase activity of ASCs and the changes in telomere length were determined. The telomerase activity of ASCs was first assessed by the expression level of TERT using quantitative RT-PCR. hTERT was undetected in both culture conditions. In telomerase assay, we observed that there was no significant difference in relative telomerase activity of ASCs when cultured in both in situ normoxia (226.59 ± 3.92) and atmospheric O2 concentration (223.67 ± 3.51) (Fig. 4B). Furthermore, no significant difference in mean TRF length was noted between ASCs cultured under both in situ normoxia (11.26 ± 0.88 kbp) and atmospheric O2 concentration (10.64 ± 0.31 kbp) (Fig. 4C).

## Discussion

ASCs at passage 3 were used in this study due to their homogeneity at this stage. Cells at earlier passage have been shown to be more heterogeneous, which contain multiple types cell such as fibroblasts, pre-adipocytes, endothelial cells and other cell types, as indicated by low expression levels of MSCs major surface markers with high expression level of haematopoetic stem cell marker CD 34 [35, 36]. To prevent haematopoetic cell contamination, which normally present in early passage and might affect the result, most studies utilized the ASCs at their homogeneous stage (eg. passage 3–6) [37–40].

Initially, characterization of ASCs was performed under both in situ normoxia and atmospheric O2 concentration to confirm that the cells used in our study were MSCs [41]. Our results demonstrated that under both conditions, ASCs displayed adherent fibroblast-like morphology. They were also able to express the major surface markers of MSCs and display adipogenic, osteogenic and chondrogenic differentiation potential, which met the minimal criteria of characterizing the human MSCs as suggested by Dominici *et al*. [34]. Additionally, the cells cultured under both conditions have also been proven to exhibit MSC functional properties (*i.e*., self-renewal properties) by CFU-F assay, as described by other studies [42–44].

Microscopic examination revealed that in situ normoxia (2% O_2_) was able to maintain the morphology of the cells. Remarkably, there was an increased density of cells under in situ normoxia as compared to atmospheric O2 concentration, suggesting that in situ normoxia increased the proliferation rate of ASCs. Moreover, ASCs culture at 2% O_2_ had an overall higher resazurin reduction and shorter population doubling time than ASCs at 21% O_2_, indicating that the cells cultured under in situ normoxia expanded more rapidly than that of atmospheric O2 concentration. Additionally, a higher cell percentage in S phase and lower in G0/G1 phase of cell cycle suggests that a larger number of cells were in the stage of DNA replication under in situ normoxia, which is in agreement with the result on cell proliferation. Our results were consistent with many studies which have shown an increased proliferation rate of ASCs at 2% O_2_ [16, 17, 24, 25]. The positive proliferative effect of ASCs under in situ normoxia suggests that this physiologically relevant condition provides a more preferable environment for their growth. It has been reported that the promotion of ASCs growth under their physiological condition might be due to the better cell adhesion and extracellular matrix formation [45] in the presence of other factors (*e.g*. growth factors) [46, 47], rather than the change in self-renewal properties of stem cells [48, 49].

Our data also demonstrate that the percentage of viable cells under in situ normoxia was higher than that of atmospheric O2 concentration with lower apoptosis rate, suggesting that in situ normoxia enhanced the survival of ASCs. Apoptosis represents a process of programmed cell death which normally occurs in response to multiple oncogenic stresses including DNA damage, chromatin changes, telomeres dysfunction and mitogenic stimulation [50]. This process is important to prevent the uncontrolled cell division that might cause neoplastic transformation [51]. The result of comet assay suggested that in situ normoxia caused less DNA damage of ASCs in comparison to atmospheric O2 concentration. This further supports the result of lower DNA damage-induced apoptosis under in situ normoxia.

Most of the studies revealed that in situ normoxia increased the viability of ASCs [16, 52, 53]. Mohyeldin *et al*. [54] demonstrated that the cells exposed to normal oxygen level are more susceptible to DNA damage than the cells cultured under in situ normoxia due to reactive oxygen species (ROS) produced by oxidative stress. Stubbs *et al*. [55] suggested that hypoxic preconditioning (<1% O_2_) increased cell viability and reduced apoptosis, whereas Valorani *et al*. [16] provided the evidence that the numbers of apoptotic cells were greater in atmospheric O2 concentration compared to that of in situ normoxia. Taken together, low oxygen tension may reduce apoptosis in response to low DNA damage.

In line with other studies [56–58], we found that HIF-1α expression was up-regulated under in situ normoxia. It has been known that when oxygen tension falls below a certain threshold, several cellular processes are regulated by HIF-1α, especially the promotion of cell proliferation and viability. This result further supports the notion that in situ normoxia promotes the survival and expansion rate of ASCs. Furthermore, we cannot exclude the possibility that growth factors might play an important role in promoting cell proliferation. In response to in situ normoxia, HIF-1α activates the expression of VEGF-A which has a very potent mitogenic characteristic [47]. FGF2 or bFGF, a member of FGF family, has also been shown to involve in cell proliferation [59]. In addition to cell proliferation, these growth factors also play a crucial role in regulating cell survival [60, 61]. Our results revealed both mRNA expression and protein secretion of VEGF-A and bFGF were significantly increased at 2% oxygen level, suggesting that these growth factors may contribute to the enhanced proliferation and viability of ASCs. Similarly, Lee *et al*. [25] suggested that in situ normoxia increased the growth rate of ASCs partly by up-regulation of VEGF and bFGF secretion. It has also been reported that under in situ normoxia, these growth factors secretion enhanced survival of the stem cells [21]. Under both culture conditions, we found that there were no significant differences in the expression of other growth factors, such as IGF, EGF, TGF, PDGF and HGF, suggesting that their production were too low, and may have little impact on cell growth and survival. Taken together, this evidence suggested that both VEGF and bFGF secreted by ASCs under in situ normoxia may play an essential role in promoting their survival and proliferation.

At low oxygen tension, the increase of ASCs proliferation ability indicates that they might undergo uncontrollable proliferation which represents one of the hallmarks of tumourigenesis [22]. Determination of tumour suppressor genes retinoblastoma (pRb), p16, p21 and p53 expression levels is a way to evaluate the tumourigenic potential of ASCs [62]. Generally, p53-p21-pRb and p16-pRb pathways are two main tumour suppressor pathways that regulate the responses of cells to oncogenic stimuli. Both pathways play a central role in regulating the progression of cell cycle and cellular senescence [63]. PRb can be activated through either or both p53-p21-pRb and p16-pRb pathways concurrently depending on the severity of stress condition. P53 tumour suppressor, a crucial regulator of cell survival, can be triggered by oxidative stress, which in turn activates its downstream target, p21. P16, another tumour suppressor, can also be induced by the same way. Up-regulation of these tumour suppressors, maintains pRb in its hypophosphorylated state, which results in cell cycle arrest [64]. It has been reported that upon DNA damage, p53 is induced to accumulate in nucleus, resulting in a higher expression level of p53. The activation of p53 in turn, would induce cell cycle arrest or apoptosis to repair damaged DNA [65].

Generally, over-expression of tumour suppressor genes, especially p53 was detected in various types of tumour cells [66, 67]. Here, we found that the expression of tumour suppressor genes pRb, p53, p21 and p16 were lower under in situ normoxia. The presence of low levels of tumor suppressor genes might be due to the low level of DNA damage which reduced apoptosis [68]. This is in agreement with other findings, suggesting that in situ normoxia reduced apoptosis in MSCs through down-regulation of p21 [69] and p16 [70]. Notably, DNA damage represents a key feature that distinguishes tumour or cancerous cells from normal cells [71]. Therefore, the low level of DNA damage observed under in situ normoxia may not raise the potential of tumourigenesis.

The tumourigenic potential of ASCs was further investigated by telomerase activity and telomere length analysis [62]. Telomerase is an enzyme involves in the maintenance of telomere length which regulates the cellular proliferative capacity [72]. One of its catalytic subunits, hTERT, acts as a template for the addition of DNA sequence to chromosomal ends [73]. During tumourigenesis, telomerase activity is activated and telomere length is stabilized, resulting in indefinite cellular proliferation [74]. Conversely, telomeres shortening might cause cellular senescence [75]. In the present study, we found that the expression of hTERT was undetected in both culture conditions, which might be due to the extremely low level of hTERT mRNA in the cells. It has been known that activation of tumour suppressor genes such as pRb, p53, p21 and p16 suppresses the expression of hTERT, whereas, the loss of those genes accelerate the telomerase activity [76, 77]. Our data show that low level of tumour suppressor genes pRb, p53, p21 and p16 under in situ normoxia did not affect the telomerase activity of the cells. We suggest that under low oxygen tension, the level of tumour suppressor genes may still adequate for hTERT suppression to maintain the basal telomerase activity of ASCs. The maintenance of low hTERT level under in situ normoxia may exclude the possibility of tumor formation. Murofushi *et al*. [78] reported that hTERT transcripts were easily detected in tumour cells but were undetectable in normal human cell lines.

In fact, there has been controversy in several research findings regarding the hypoxic effect on telomere length and telomerase activity of ASCs. Dos Santos *et al*. [79] found that expanding bone marrow mesenchymal stem cells (BMMCs) under low oxygen tension showed a greater shortening of telomere length in relation to their high proliferation rate with undetermined telomerase activity. In contrast, Yanada *et al*. [80], suggested that with the presence of bFGF, MSCs enhance their proliferation without affecting their telomerase activity while reducing their telomere shortening rate. However, the mechanism involved has yet to be determined. Our data indicate that under in situ normoxia, the telomerase activity of ASCs was maintained with normal telomere shortening rate despite their high proliferative ability. This result was consistent with the findings of Buchheiser *et al*. [81], suggesting that under low oxygen tension, MSCs was able to proliferate rapidly without up-regulating their telomerase activity. Their telomere shortening rate was maintained with an undefined mechanism. Taken together, the unaffected telomere length and telomerase activity by low oxygen tension suggests that in situ normoxia does not increase the potential of developing tumour.

## Conclusion

In this paper, we have studied the tumourigenic potential of human ASCs under both in situ normoxia and atmospheric O2 concentration. Our findings clearly showed that expansion of ASCs under 2% O_2_ did not increase the risk of tumourigenesis even they possess a high proliferative ability and survival rate. These characteristics might be correlated to the upregulation of HIF-1α and its downstream targets, VEGF-A and bFGF. We suggest that the rapid growth of ASCs under this oxygen tension is kept under control without reaching the stage of tumourigenesis. Since there is low risk of tumourigenicity in this study, further studies involving tumourigenic assessment on long term expansion of ASCs at their physiological condition may offer greater insight into the safe use of ASCs in clinical therapy. Given the urgent need for the production of large quantities of high quality cells in the clinical field, growing ASCs under this physiological condition may prove to be an important option for clinical therapy.

## Supporting Information

S1 Fig In situ normoxia did not alter the phenotype of ASCs. Representative histograms by flow cytometry analysis for both atmospheric O2 concentration (A) and in situ normoxia (B). Graph shows the percentage of expression of ASCs surface markers (C). In situ normoxia maintained the ASCs surface markers expression CD73, CD90, CD105 and HLA ABC.
(TIF)

S2 Fig ASCs maintained their differentiation abilities under in situ normoxia. Adipogenic differentiation of ASCs was confirmed by Oil Red O staining, magnification 400x. Arrows show the formation of lipid droplets (A). Osteogenic differentiation of ASCs was detected by Alizarin Red staining, magnification 100x. Arrows show the formation of calcium deposits (B). Alcian blue staining was used to confirm the chondrogenic differentiation of ASCs, magnification 40x. The accumulations of proteoglycans are indicated by arrows (C).
(TIF)

## References

[1] Smith A (2006) A glossary for stem-cell biology. Nature 441: 1060–1060. 10.1038/nature04954

[2] Doulatov S, Daley GQ (2013) A Stem Cell Perspective on Cellular Engineering. Science 342: 700–702. 10.1126/science.1238363 24202165

[3] Wagers AJ (2012) The Stem Cell Niche in Regenerative Medicine. Cell Stem Cell 10: 362–369. 10.1016/j.stem.2012.02.018 22482502

[4] Rubin JP, Marra KG (2013) Adipose stem cell therapy for soft tissue reconstruction. Lancet 382: 1077–1079. 10.1016/S0140-6736(13)61744-4 24075037

[5] Kolle SF, Fischer-Nielsen A, Mathiasen AB, Elberg JJ, Oliveri RS, et al (2013) Enrichment of autologous fat grafts with ex-vivo expanded adipose tissue-derived stem cells for graft survival: a randomised placebo-controlled trial. Lancet 382: 1113–1120. 10.1016/S0140-6736(13)61410-5 24075051

[6] Schäffler A, Büchler C (2007) Concise Review: Adipose Tissue-Derived Stromal Cells-Basic and Clinical Implications for Novel Cell-Based Therapies. Stem Cells 25: 818–827. 10.1634/stemcells.2006-0589 17420225

[7] Crisan M, Casteilla L, Lehr L, Carmona M, Paoloni-Giacobino A, et al (2008) A Reservoir of Brown Adipocyte Progenitors in Human Skeletal Muscle. Stem Cells 26: 2425–2433. 10.1634/stemcells.2008-0325 18617684

[8] Kim JH, Park SH, Park SG, Choi JS, Xia Y, et al (2011) The pivotal role of reactive oxygen species generation in the hypoxia-induced stimulation of adipose-derived stem cells. Stem Cells Dev 20: 1753–1761. 10.1089/scd.2010.0469 21265612 PMC3182032

[9] Traktuev DO, Merfeld-Clauss S, Li J, Kolonin M, Arap W, et al (2008) A Population of Multipotent CD34-Positive Adipose Stromal Cells Share Pericyte and Mesenchymal Surface Markers, Reside in a Periendothelial Location, and Stabilize Endothelial Networks. Circulation Research 102: 77–85. 10.1161/CIRCRESAHA.107.159475 17967785

[10] Matsumoto A, Matsumoto S, Sowers AL, Koscielniak JW, Trigg NJ, et al (2005) Absolute oxygen tension (pO2) in murine fatty and muscle tissue as determined by EPR. Magnetic Resonance in Medicine 54: 1530–1535. 10.1002/mrm.20714 16276490

[11] Pasarica M, Sereda OR, Redman LM, Albarado DC, Hymel DT, et al (2008) Reduced Adipose Tissue Oxygenation in Human Obesity: Evidence for Rarefaction, Macrophage Chemotaxis, and Inflammation Without an Angiogenic Response. Diabetes 58: 718–725. 10.2337/db08-1098 19074987 PMC2646071

[12] Chung H-M, Won C-H, Sung J-H (2009) Responses of adipose-derived stem cells during hypoxia- enhanced skin-regenerative potential. Expert Opin Biol Ther 9: 1499–1508. 10.1517/14712590903307362 19780713

[13] Scadden DT (2006) The stem-cell niche as an entity of action. Nature 441: 1075–1079. 10.1038/nature04957 16810242

[14] Ivanovic Z (2009) Hypoxia or in situ normoxia: The stem cell paradigm. J Cell Physiol 219: 271–275. 10.1002/jcp.21690 19160417

[15] Estrada JC, Albo C, Benguría A, Dopazo A, López-Romero P, et al (2011) Culture of human mesenchymal stem cells at low oxygen tension improves growth and genetic stability by activating glycolysis. Cell Death and Differentiation 19: 743–755. 10.1038/cdd.2011.172 22139129 PMC3321628

[16] Valorani MG, Montelatici E, Germani A, Biddle A, D′Alessandro D, et al (2012) Pre-culturing human adipose tissue mesenchymal stem cells under hypoxia increases their adipogenic and osteogenic differentiation potentials. Cell Proliferation 45: 225–238. 10.1111/j.1365-2184.2012.00817.x 22507457 PMC6622217

[17] Xu Y, Malladi P, Chiou M, Bekerman E, Giaccia AJ, et al (2007) In vitro expansion of adipose-derived adult stromal cells in hypoxia enhances early chondrogenesis. Tissue Eng 13: 2981–2993. 10.1089/ten.2007.0050 17916040

[18] Hung SP, Yang MH, Tseng KF, Lee OK (2013) Hypoxia-induced secretion of TGF-beta1 in mesenchymal stem cell promotes breast cancer cell progression. Cell Transplant 22: 1869–1882. 10.3727/096368912X657954 23067574

[19] Thangarajah H, Vial IN, Chang E, El-Ftesi S, Januszyk M, et al (2009) IFATS Collection: Adipose Stromal Cells Adopt a Proangiogenic Phenotype Under the Influence of Hypoxia. Stem Cells 27: 266–274. 10.1634/stemcells.2008-0276 18974212

[20] Kim S, Chaudhry A, Lee I, Frank JA (2013) Effects of Long-Term Hypoxia and Pro-Survival Cocktail in Bone Marrow-Derived Stromal Cell Survival. Stem Cells Dev 20: 20. 10.1089/scd.2013.0297 PMC3928716 24147599

[21] Crisostomo PR, Wang Y, Markel TA, Wang M, Lahm T, et al (2008) Human mesenchymal stem cells stimulated by TNF-α, LPS, or hypoxia produce growth factors by an NFκB- but not JNK-dependent mechanism. American Journal of Physiology—Cell Physiology 294: C675–C682. 10.1152/ajpcell.00437.2007 18234850

[22] Stoeltzing O, McCarty MF, Wey JS, Fan F, Liu W, et al (2004) Role of Hypoxia-Inducible Factor 1 in Gastric Cancer Cell Growth, Angiogenesis, and Vessel Maturation. JNCI Journal of the National Cancer Institute 96: 946–956. 10.1093/jnci/djh168 15199114

[23] Hanahan D, Weinberg RA (2011) Hallmarks of Cancer: The Next Generation. Cell 144: 646–674. 10.1016/j.cell.2011.02.013 21376230

[24] Valorani MG, Germani A, Otto WR, Harper L, Biddle A, et al (2010) Hypoxia increases Sca-1/CD44 co-expression in murine mesenchymal stem cells and enhances their adipogenic differentiation potential. Cell Tissue Res 341: 111–120. 10.1007/s00441-010-0982-8 20496083

[25] Lee EY, Xia Y, Kim WS, Kim MH, Kim TH, et al (2009) Hypoxia-enhanced wound-healing function of adipose-derived stem cells: increase in stem cell proliferation and up-regulation of VEGF and bFGF. Wound Repair Regen 17: 540–547. 10.1111/j.1524-475X.2009.00499.x 19614919

[26] Choi JR, Pingguan-Murphy B, Wan Abas WAB, Noor Azmi MA, Omar SZ, et al (2014) Impact of low oxygen tension on stemness, proliferation and differentiation potential of human adipose-derived stem cells. Biochemical and Biophysical Research Communications 448: 218–224. 10.1016/j.bbrc.2014.04.096 24785372

[27] Rosland GV, Svendsen A, Torsvik A, Sobala E, McCormack E, et al (2009) Long-term cultures of bone marrow-derived human mesenchymal stem cells frequently undergo spontaneous malignant transformation. Cancer Res 69: 5331–5339. 10.1158/0008-5472.CAN-08-4630 19509230

[28] Rubio D, Garcia-Castro J, Martin MC, de la Fuente R, Cigudosa JC, et al (2005) Spontaneous human adult stem cell transformation. Cancer Res 65: 3035–3039. 15833829 10.1158/0008-5472.CAN-04-4194

[29] Barkholt L, Flory E, Jekerle V, Lucas-Samuel S, Ahnert P, et al (2013) Risk of tumorigenicity in mesenchymal stromal cell–based therapies—Bridging scientific observations and regulatory viewpoints. Cytotherapy 15: 753–759. 10.1016/j.jcyt.2013.03.005 23602595

[30] Pan Q, Fouraschen SM, de Ruiter PE, Dinjens WN, Kwekkeboom J, et al. (2013) Detection of spontaneous tumorigenic transformation during culture expansion of human mesenchymal stromal cells. Experimental Biology and Medicine: 1535370213506802. 10.1177/1535370213506802 24227633

[31] Ben-David U, Mayshar Y, Benvenisty N (2011) Large-scale analysis reveals acquisition of lineage-specific chromosomal aberrations in human adult stem cells. Cell Stem Cell 9: 97–102. 10.1016/j.stem.2011.06.013 21816361

[32] Prockop DJ, Keating A (2012) Relearning the Lessons of Genomic Stability of Human Cells During Expansion in Culture: Implications for Clinical Research. STEM CELLS 30: 1051–1052. 10.1002/stem.1103 22495826

[33] Wan Kamarul Zaman WS, Makpol S, Sathapan S, Chua KH (2014) Long-term in vitro expansion of human adipose-derived stem cells showed low risk of tumourigenicity. Journal of Tissue Engineering and Regenerative Medicine 8: 67–76. 10.1002/term.1501 22552847

[34] Dominici M, Le Blanc K, Mueller I, Slaper-Cortenbach I, Marini F, et al (2006) Minimal criteria for defining multipotent mesenchymal stromal cells. The International Society for Cellular Therapy position statement. Cytotherapy 8: 315–317. 16923606 10.1080/14653240600855905

[35] Mitchell JB, McIntosh K, Zvonic S, Garrett S, Floyd ZE, et al (2006) Immunophenotype of Human Adipose-Derived Cells: Temporal Changes in Stromal-Associated and Stem Cell-Associated Markers. Stem Cells 24: 376–85. 10.1634/stemcells.2005-0234 16322640

[36] Sidney LE, Branch MJ, Dunphy SE, Dua HS, Hopkinson A (2014) Concise Review: Evidence for CD34 as a Common Marker for Diverse Progenitors. Stem Cells 32: 1380–1389. 10.1002/stem.1661 24497003 PMC4260088

[37] Hsiao ST, Lokmic Z, Peshavariya H, Abberton KM, Dusting GJ, et al (2013) Hypoxic conditioning enhances the angiogenic paracrine activity of human adipose-derived stem cells. Stem cells and development 22: 1614–1623. 10.1089/scd.2012.0602 23282141 PMC3653395

[38] Sheehy EJ, Buckley CT, Kelly DJ (2012) Oxygen tension regulates the osteogenic, chondrogenic and endochondral phenotype of bone marrow derived mesenchymal stem cells. Biochemical and biophysical research communications 417: 305–310. 10.1016/j.bbrc.2011.11.105 22155244

[39] Dos Santos F, Andrade PZ, Boura JS, Abecasis MM, Da Silva CL, et al (2010) Ex vivo expansion of human mesenchymal stem cells: a more effective cell proliferation kinetics and metabolism under hypoxia. Journal of cellular physiology 223: 27–35. 20020504 10.1002/jcp.21987

[40] Malladi P, Xu Y, Chiou M, Giaccia AJ, Longaker MT (2006) Effect of reduced oxygen tension on chondrogenesis and osteogenesis in adipose-derived mesenchymal cells. American Journal of Physiology-Cell Physiology 290: C1139–C1146. 10.1152/ajpcell.00415.2005 16291817

[41] Charbord P, Casteilla L (2011) [Human mesenchymal stem cell biology]. Med Sci (Paris) 27: 261–267. 10.1051/medsci/2011273261 21447295

[42] Wagegg M, Gaber T, Lohanatha FL, Hahne M, Strehl C, et al (2012) Hypoxia promotes osteogenesis but suppresses adipogenesis of human mesenchymal stromal cells in a hypoxia-inducible factor-1 dependent manner. PLoS One 7: e46483 10.1371/journal.pone.0046483 23029528 PMC3459928

[43] Berniakovich I, Giorgio M (2013) Low Oxygen Tension Maintains Multipotency, Whereas Normoxia Increases Differentiation of Mouse Bone Marrow Stromal Cells. International Journal of Molecular Sciences 14: 2119–2134. 10.3390/ijms14012119 23340651 PMC3565369

[44] Wagner W, Wein F, Seckinger A, Frankhauser M, Wirkner U, et al (2005) Comparative characteristics of mesenchymal stem cells from human bone marrow, adipose tissue, and umbilical cord blood. Exp Hematol 33: 1402–1416. 10.1016/j.exphem.2005.07.003 16263424

[45] Grayson WL, Zhao F, Bunnell B, Ma T (2007) Hypoxia enhances proliferation and tissue formation of human mesenchymal stem cells. Biochemical and Biophysical Research Communications 358: 948–953. 10.1016/j.bbrc.2007.05.054 17521616

[46] Ahn HJ, Lee WJ, Kwack K, Kwon YD (2009) FGF2 stimulates the proliferation of human mesenchymal stem cells through the transient activation of JNK signaling. FEBS Lett 583: 2922–2926. 10.1016/j.febslet.2009.07.056 19664626

[47] Chen G, Shi X, Sun C, Li M, Zhou Q, et al (2013) VEGF-mediated proliferation of human adipose tissue-derived stem cells. PLoS One 8: e73673 10.1371/journal.pone.0073673 24098328 PMC3789739

[48] Tamama K, Kawasaki H, Kerpedjieva SS, Guan J, Ganju RK, et al (2011) Differential roles of hypoxia inducible factor subunits in multipotential stromal cells under hypoxic condition. J Cell Biochem 112: 804–817. 10.1002/jcb.22961 21328454 PMC4110974

[49] Sheehy EJ, Buckley CT, Kelly DJ (2012) Oxygen tension regulates the osteogenic, chondrogenic and endochondral phenotype of bone marrow derived mesenchymal stem cells. Biochem Biophys Res Commun 417: 305–310. 10.1016/j.bbrc.2011.11.105 22155244

[50] Roos WP, Kaina B (2006) DNA damage-induced cell death by apoptosis. Trends Mol Med 12: 440–450. 10.1016/j.molmed.2006.07.007 16899408

[51] Plotkin JB, Nowak MA (2002) The Different Effects of Apoptosis and DNA Repair on Tumorigenesis. Journal of Theoretical Biology 214: 453–467. 10.1006/jtbi.2001.2471 11846602

[52] Pilgaard L, Lund P, Duroux M, Lockstone H, Taylor J, et al (2009) Transcriptional signature of human adipose tissue-derived stem cells (hASCs) preconditioned for chondrogenesis in hypoxic conditions. Experimental Cell Research 315: 1937–1952. 10.1016/j.yexcr.2009.01.020 19331821

[53] Rehman J (2004) Secretion of Angiogenic and Antiapoptotic Factors by Human Adipose Stromal Cells. Circulation 109: 1292–1298. 10.1161/01.CIR.0000121425.42966.F1 14993122

[54] Mohyeldin A, Garzón-Muvdi T, Quiñones-Hinojosa A (2010) Oxygen in Stem Cell Biology: A Critical Component of the Stem Cell Niche. Cell Stem Cell 7: 150–161. 10.1016/j.stem.2010.07.007 20682444

[55] Stubbs SL, Hsiao ST-F, Peshavariya HM, Lim SY, Dusting GJ, et al (2012) Hypoxic Preconditioning Enhances Survival of Human Adipose-Derived Stem Cells and Conditions Endothelial Cells In Vitro. Stem Cells and Development 21: 1887–1896. 10.1089/scd.2011.0289 22165914

[56] Shih-Chieh H, Radhika RP, Shu-Ching H, Cecelia S, Sy-Chi C, et al (2007) Short-Term Exposure of Multipotent Stromal Cells to Low Oxygen Increases Their Expression of CX3CR1 and CXCR4 and Their Engraftment In Vivo. PLoS ONE 2. 10.1371/journal.pone.0000416 PMC1855077 17476338

[57] Yang D-C, Yang M-H, Tsai C-C, Huang T-F, Chen Y-H, et al (2011) Hypoxia Inhibits Osteogenesis in Human Mesenchymal Stem Cells through Direct Regulation of RUNX2 by TWIST. PLoS ONE 6: e23965 10.1371/journal.pone.0023965 21931630 PMC3170288

[58] Wagegg M, Gaber T, Lohanatha FL, Hahne M, Strehl C, et al (2012) Hypoxia Promotes Osteogenesis but Suppresses Adipogenesis of Human Mesenchymal Stromal Cells in a Hypoxia-Inducible Factor-1 Dependent Manner. PLoS ONE 7: e46483 10.1371/journal.pone.0046483 23029528 PMC3459928

[59] Ahn H-J, Lee W-J, Kwack K, Kwon YD (2009) FGF2 stimulates the proliferation of human mesenchymal stem cells through the transient activation of JNK signaling. FEBS Letters 583: 2922–2926. 10.1016/j.febslet.2009.07.056 19664626

[60] Byrne AM, Bouchier-Hayes DJ, Harmey JH (2005) Angiogenic and cell survival functions of vascular endothelial growth factor (VEGF). J Cell Mol Med 9: 777–794. 10.1111/j.1582-4934.2005.tb00379.x 16364190 PMC6740098

[61] Eiselleova L, Matulka K, Kriz V, Kunova M, Schmidtova Z, et al (2009) A complex role for FGF-2 in self-renewal, survival, and adhesion of human embryonic stem cells. Stem Cells 27: 1847–1857. 10.1002/stem.128 19544431 PMC2798073

[62] Wan Kamarul Zaman WS, Makpol S, Sathapan S, Chua KH (2012) Long-term in vitro expansion of human adipose-derived stem cells showed low risk of tumourigenicity. Journal of Tissue Engineering and Regenerative Medicine 8: 67–76. 10.1002/term.1501 22552847

[63] Pelicci PG (2004) Do tumor-suppressive mechanisms contribute to organism aging by inducing stem cell senescence? J Clin Invest 113: 4–7. 10.1172/JCI200420750 14702099 PMC300887

[64] Lilian C-N, Magda Carolina S-C, Sandra Rocío R-C (2010) The Dual Role of Senescence in Tumorigenesis. Sociedad Chilena de Anatomía 28.

[65] Solozobova V, Rolletschek A, Blattner C (2009) Nuclear accumulation and activation of p53 in embryonic stem cells after DNA damage. BMC Cell Biology 10: 46 10.1186/1471-2121-10-46 19534768 PMC2704172

[66] Inoue K, Kurabayashi A, Shuin T, Ohtsuki Y, Furihata M (2012) Overexpression of p53 protein in human tumors. Medical Molecular Morphology 45: 115–123. 10.1007/s00795-012-0575-6 23001293

[67] Kikuchi S, Nishimura R, Osako T, Okumura Y, Nishiyama Y, et al (2013) Definition of p53 overexpression and its association with the clinicopathological features in luminal/HER2-negative breast cancer. Anticancer Res 33: 3891–3897. 24023325

[68] Offer H, Erez N, Zurer I, Tang X, Milyavsky M, et al (2002) The onset of p53-dependent DNA repair or apoptosis is determined by the level of accumulated damaged DNA. Carcinogenesis 23: 1025–1032. 10.1093/carcin/23.6.1025 12082025

[69] Tsai CC, Chen YJ, Yew TL, Chen LL, Wang JY, et al (2010) Hypoxia inhibits senescence and maintains mesenchymal stem cell properties through down-regulation of E2A-p21 by HIF-TWIST. Blood 117: 459–469. 10.1182/blood-2010-05-287508 20952688

[70] Jin Y, Kato T, Furu M, Nasu A, Kajita Y, et al (2010) Mesenchymal stem cells cultured under hypoxia escape from senescence via down-regulation of p16 and extracellular signal regulated kinase. Biochem Biophys Res Commun 391: 1471–1476. 10.1016/j.bbrc.2009.12.096 20034468

[71] Gorgoulis VG, Vassiliou LV, Karakaidos P, Zacharatos P, Kotsinas A, et al (2005) Activation of the DNA damage checkpoint and genomic instability in human precancerous lesions. Nature 434: 907–913. 10.1038/nature03485 15829965

[72] Gomez DE, Armando RG, Farina HG, Menna PL, Cerrudo CS, et al (2012) Telomere structure and telomerase in health and disease (review). Int J Oncol 41: 1561–1569. 10.3892/ijo.2012.1611 22941386 PMC3583695

[73] Blasco MaA (2003) Telomeres and cancer: a tale with many endings. Current Opinion in Genetics & Development 13: 70–76. 10.1016/S0959-437X(02)00011-4 12573438

[74] Artandi SE, DePinho RA (2010) Telomeres and telomerase in cancer. Carcinogenesis 31: 9–18. 10.1093/carcin/bgp268 19887512 PMC3003493

[75] Stewart SA, Weinberg RA (2002) Senescence: does it all happen at the ends? Oncogene 21: 627–630. 10.1038/sj.onc.1205062 11850788

[76] Stampfer MR, Garbe J, Nijjar T, Wigington D, Swisshelm K, et al (2003) Loss of p53 function accelerates acquisition of telomerase activity in indefinite lifespan human mammary epithelial cell lines. Oncogene 22: 5238–5251. 10.1038/sj.onc.1206667 12917625

[77] Cong YS, Wright WE, Shay JW (2002) Human telomerase and its regulation. Microbiol Mol Biol Rev 66: 407–425. 10.1128/MMBR.66.3.407-425.2002 12208997 PMC120798

[78] Murofushi Y, Nagano S, Kamizono J, Takahashi T, Fujiwara H, et al (2006) Cell cycle-specific changes in hTERT promoter activity in normal and cancerous cells in adenoviral gene therapy: a promising implication of telomerase-dependent targeted cancer gene therapy. Int J Oncol 29: 681–688. 16865285

[79] Dos Santos F, Andrade PZ, Boura JS, Abecasis MM, da Silva CL, et al (2010) Ex vivo expansion of human mesenchymal stem cells: a more effective cell proliferation kinetics and metabolism under hypoxia. J Cell Physiol 223: 27–35. 20020504 10.1002/jcp.21987

[80] Yanada S, Ochi M, Kojima K, Sharman P, Yasunaga Y, et al (2006) Possibility of selection of chondrogenic progenitor cells by telomere length in FGF-2-expanded mesenchymal stromal cells. Cell Prolif 39: 575–584. 10.1111/j.1365-2184.2006.00397.x 17109640 PMC6496787

[81] Buchheiser A, Houben AP, Bosch J, Marbach J, Liedtke S, et al (2012) Oxygen tension modifies the ‘stemness’ of human cord blood-derived stem cells. Cytotherapy 14: 967–982. 10.3109/14653249.2012.671518 22494073

