# Supplementary figures and images for: In Situ Normoxia Enhances Survival and Proliferation Rate of Human Adipose Tissue-Derived Stromal Cells without Increasing the Risk of Tumourigenesis
Source: PLoS One. 2015 Jan 23;10(1):e0115034. doi: 10.1371/journal.pone.0115034 (PMC4304807; doi:10.1371/journal.pone.0115034)

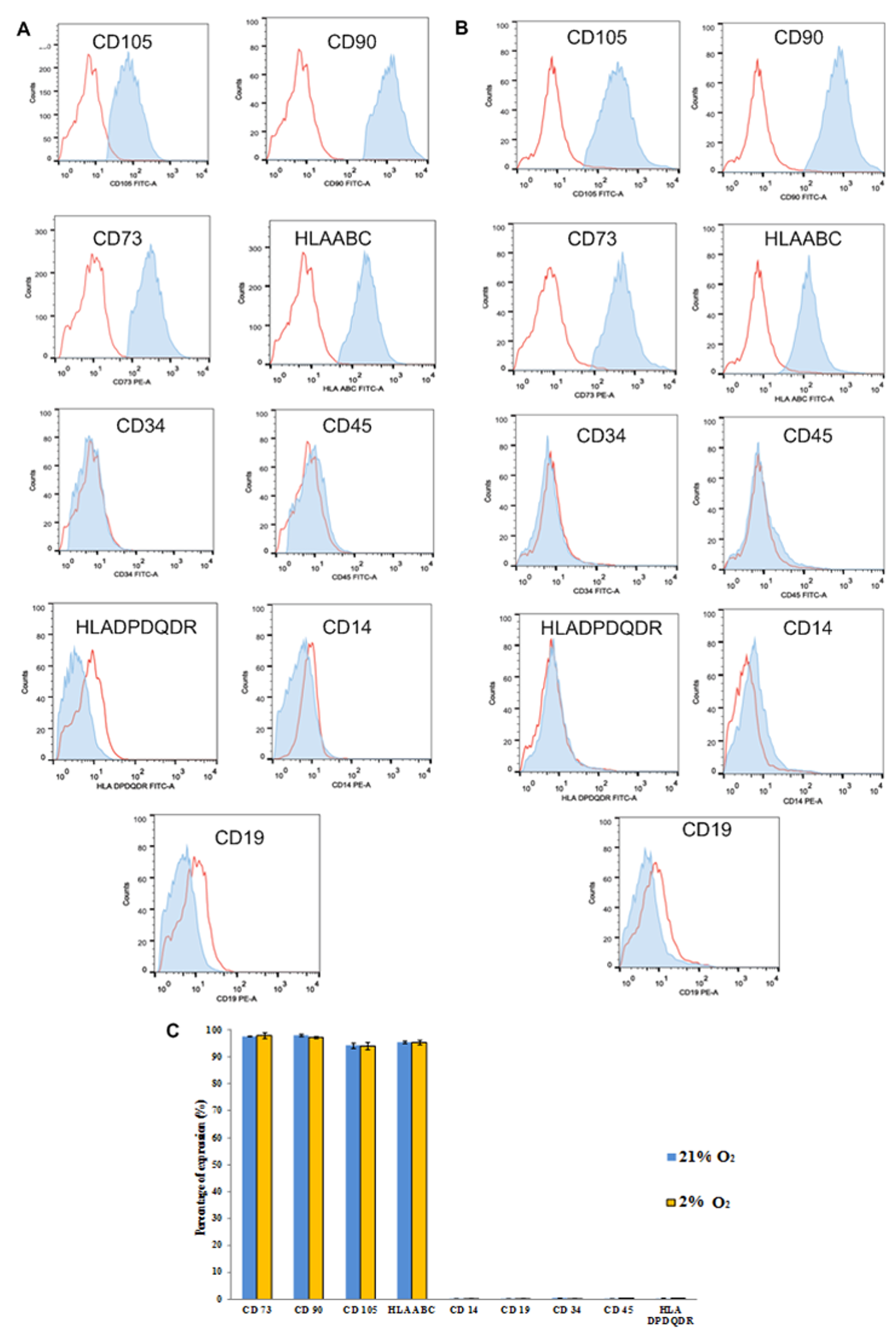

Supplement: S1 Fig — Representative histograms by flow cytometry analysis for both atmospheric O2 concentration (A) and in situ normoxia (B). Graph shows the percentage of expression of ASCs surface markers (C). In situ normoxia maintained the ASCs surface markers expression CD73, CD90, CD105 and HLA ABC. (TIF) [file pone.0115034.s001.tif]

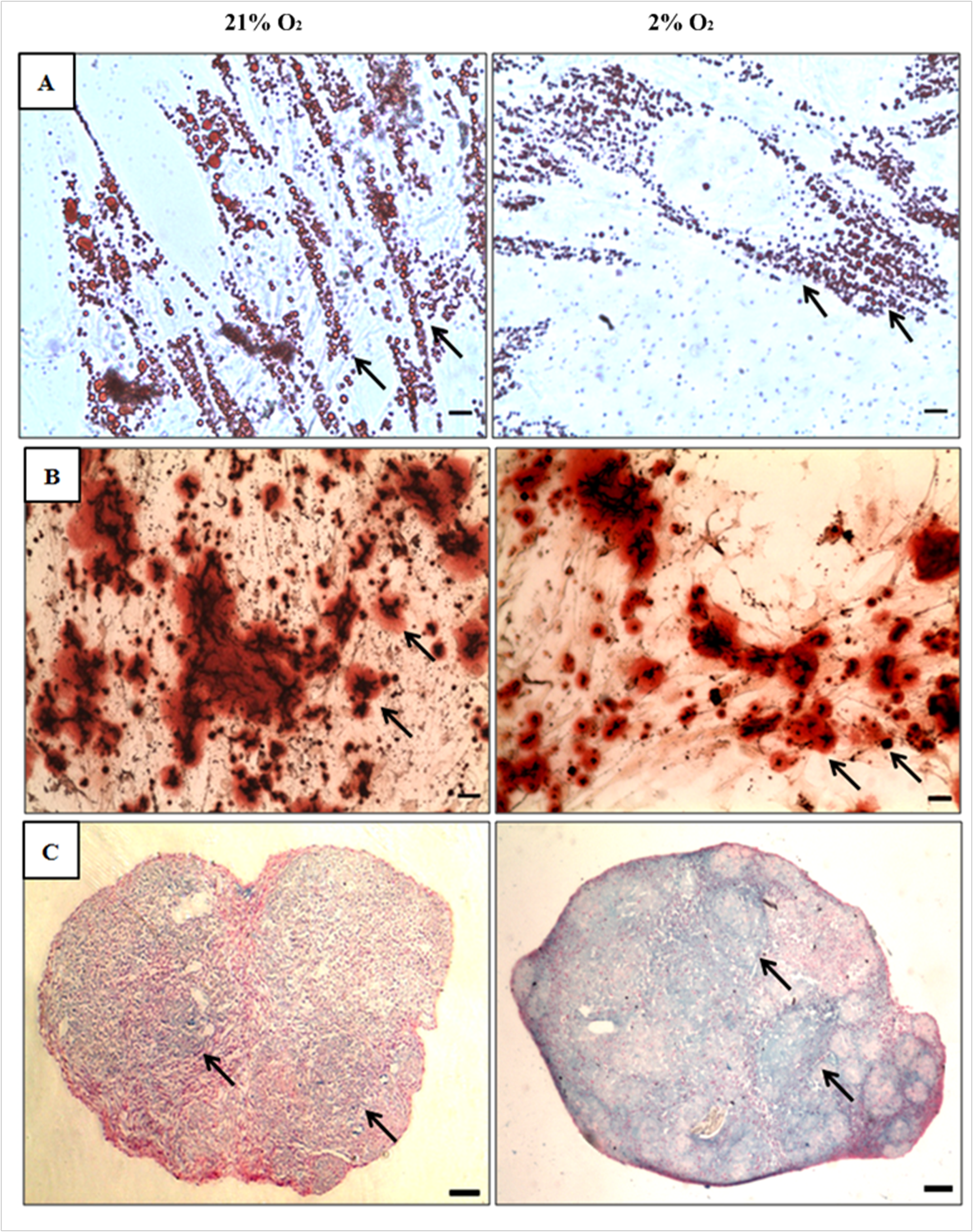

Supplement: S2 Fig — Adipogenic differentiation of ASCs was confirmed by Oil Red O staining, magnification 400x. Arrows show the formation of lipid droplets (A). Osteogenic differentiation of ASCs was detected by Alizarin Red staining, magnification 100x. Arrows show the formation of calcium deposits (B). Alcian blue staining was used to confirm the chondrogenic differentiation of ASCs, magnification 40x. The accumulations of proteoglycans are indicated by arrows (C). (TIF) [file pone.0115034.s002.tif]
